# The Fight Against *Panax notoginseng* Root-Rot Disease Using Zingiberaceae Essential Oils as Potential Weapons

**DOI:** 10.3389/fpls.2018.01346

**Published:** 2018-10-04

**Authors:** Yan-Jiao Yin, Chuan-Jiao Chen, Shi-Wei Guo, Ke-Ming Li, Yu-Nan Ma, Wu-Mei Sun, Fu-Rong Xu, Yong-Xian Cheng, Xian Dong

**Affiliations:** ^1^College of Pharmaceutical Sciences, Yunnan University of Traditional Chinese Medicine, Kunming, China; ^2^College of Resources and Environmental Science, Nanjing Agricultural University, Nanjing, China; ^3^Guangdong Key Laboratory for Genome Stability and Disease Prevention, School of Pharmaceutical Sciences, Shenzhen University Health Science Center, Shenzhen, China

**Keywords:** *Panax notoginseng*, root-rot disease, Zingiberaceae, EOs, fungi

## Abstract

The root of *Panax notoginseng* (*P. notoginseng*) is one of the most highly valuable medicinal herbs in China owing to its pronounced hemostatic and restorative properties. Despite this important fact, growing *P. notoginseng* is seriously limited by root-rot diseases. In studies aimed at developing a solution to this problem, environment-friendly essential oils (EOs) of five medicinal plants of the family Zingiberaceae were tested for their inhibitory effects on the growth of three main soil pathogens associated with the root-rot diseases of *P. notoginseng*. The results showed that the EOs of *Alpinia katsumadai* Hayata and *Zingiber officinale* Roscoe promote significant reductions in the mycelium growth of the pathogen *in vitro* at a concentration of 50 mg mL^−1^, which is much higher than that needed (5 mg mL^−1^) to reduce growth by the positive control, flutriafol. Furthermore, the chemical components of the two EOs were determined by using GC-MS analysis. Eucalyptol was found to account for more than 30% of the oils of the two plants, with the second major components being geranyl acetate and α-terpineol. These substances display different degrees of fungistasis *in vitro*. To further determine the effects of the EO of *Zingiber officinale (Z. officinale) in vivo*, soilless cultivation of *P. notoginseng* with pathogen inoculation was conducted in a greenhouse. Addition of the petroleum ether extract (approximately equal to EO) of *Z. officinale* to the culture matrix causes a large decrease in both the occurrence and severity of the *P. notoginseng* root-rot disease. The decreasing trend of net photosynthetic rate (P_n_), stomatal conductance (g_s_), intercellular CO_2_ concentration (C_i_), and transpiration rate (T_r_) were all alleviated. In addition, the activities of catalase (CAT), peroxidase (POD), and the malondialdehyde (MDA) content were also largely reduced after pathogen infection, with the root activity being higher than that of the control. Taken together, the findings reveal that the EOs from plants might serve as promising sources of eco-friendly natural pesticides with less chemical resistance.

## Introduction

Soilless cultivation is a method that employs a matrix or substrate only, instead of a natural soil for seedling cultivation and utilizes irrigation with nutrient solutions after planting. This new approach to cultivation was performed initially in China and has since then developed rapidly. In 2011, it was applied for the production of field crops, fruits, vegetables, and flowers over a total area of more than 3,000 hm^2^. Soilless cultivation has great potential to be used for future modernization in agriculture (Wang Z. et al., 2013). The major advantage of this technique is that it uncouples plant growth from problems associated with the soil. Also, the regulation of plant nutrients via irrigation could manage the delivery of nutrients to plants.

At present, soil-borne diseases of medical plants have spread rapidly in many areas. Although rotation is the main method utilized to prevent these types of diseases, the narrow growth environment of medical plants prevents the implementation of this method because it increases planting costs and seriously affects the quality and yields of the products, both of which affect the development of the biopharmaceutical industry. Also, the use of a large number of pesticides, which are difficult to control, causes a vicious cycle of ecological flora imbalance and the deposition of pesticide residues and soil pollutants (Zhang et al., 2012). Searching for ways to prevent and control root-rot diseases has encountered a number of problems over the years.

*Panax notoginseng* is a perennial herb of the family Araliaceae, whose root is used as a medicinal herb in China. The recent pharmacological and clinical studies revealed that the root of *P. notoginseng* has significant pharmacological effects, especially on the prevention and treatment of cardiovascular and cerebrovascular diseases (Yao, 2002). However, *P. notoginseng* no longer exists in the wild state because of the environmental changes and large-scale uncontrolled excavation. Cultivation of *P. notoginseng* is mainly carried out in Wenshan, in the Yunnan province of China (Kan et al., 2016), where the yields and quality are the highest in the world (Wang Y. et al., 2013). Since its growth needs perennial shade and a warm, damp environment, intensive planting of *P. notoginseng* over large areas could easily lead to epidemic levels of pests and diseases.

Among the diseases impacting *P. notoginseng*, root-rot is the most common and difficult to control (Luo et al., 1997). Root-rot typically occurs in yearling *P. notoginseng* or in two-year-old *P. notoginseng* plants, where the disease is more severe. Pathogen infection is followed by wilting and yellowing of leaves and decaying of the underground parts. The average annual loss caused by root-rot is 5%~20%, and it could increase up to 70% when the disease becomes more severe (Mao et al., 2013).

As a result of the impact described above, studies on *P. notoginseng* root-rot disease have increased significantly. In 1991, Cao and Qi (1991) proposed that *P. notoginseng* root-rot disease is caused by the specific mycorrhizal type, *Fusarium solani*. Later, Wang et al. (2015) suggested that *Fusarium oxysporum* E. F. Sm. and Swingle, and *Fusarium monilliforme* var. *intermedium* Neish and M. Legg are the the main pathogens responsible for this disease. Adding further complexity to the issue, a study by Miao et al. indicated that *Cylindrocarpon destructans* is an important pathogen of *P. notoginseng* root-rot disease (Miao et al., 2006). Due to the variety of proposals, the prevention and treatment of *P. notoginseng* root-rot disease has not been straightforward. In general, continuous cropping disorders of *P. notoginseng* can affect the diversity of soil microbial communities (Wang Y. et al., 2013; Li et al., 2017; Liu et al., 2018), and the soil microbial communities are influenced by multiple factors such as plant type, climate, soil properties, and agricultural practice (Li et al., 2017). Thus, it is possible for many sources of this soil-borne disease to exist.

In recent years, botanical pesticides have been a research topic of great interest because active components extracted from plants can inhibit bacteria and fungi. The results of efforts in this area have shown that the essential oils (EOs) of the plant family Zingiberaceae display strong inhibitory activities against some bacteria, including *Staphylococcus aureus* (Sivasothy et al., 2011) and fungi, such as *Aspergillus niger* (Sasidharan and Menon, 2010). Zingiberaceae is composed of about 53 genera and 1200 species, as exemplified by *Alpinia katsumadai* Hayata and *Alpinia oxyphylla* Miq. *Zingiber* which belong to *Alpinia*; *Zingiber officinale* Roscoe which belongs to *Zingiber*; *Kaempferia galanga* L., which belongs to *Kaempferia;* and *Curcuma Longa* L., which belongs to *Curcuma*. Many Zingiberaceae plant species are used as herbs and for flavoring. The main secondary metabolites of Zingiberaceae are polysaccharides, flavonoids, and EOs (Kress et al., 2002; Tushar et al., 2010). As safe substitutes for synthetic pesticides, the use of EOs will lead to a reduction in environmental pollutants and could play an important role in the prevention and control of root rot (Hashem et al., 2010). In the present study, we explored the soilless cultivation of *P. notoginseng*, and the antifungal properties of EOs from five kinds of Zingiberaceae plants species against three species of *P. notoginseng* root-rot pathogens were studied. The aim of the effort was to assess the effects of using EOs as biological pesticides in combination with the soilless cultivation method by measuring several *P. notoginseng*-related factors. In the investigation we specifically determined the effect on *P. notoginseng* plant growth, disease index, disease incidence, photosynthesis, and the activities of catalase (CAT), peroxidase (POD) and the malondialdehyde (MDA) content in order to evaluate how root-rot disease can be prevented without requiring treatments that result in environment pollution.

## Materials and methods

### Plant cultivation

*P. notoginseng* seeds were seeded in a 1:2 mixture of quartz sand and roseite in a greenhouse. After germination, the seedlings were cultivated under 20°C with a relative humidity of 70 ± 10% and a photoperiod of 14 h during daytime. The seedlings were supplied with a full-strength Hoagland nutrient solution. The macronutrient composition of the Hoagland nutrient solution (in mg L^−1^) includes 40 N (NH_4_NO_3_), 10 P (KH_2_PO_4_), 40 K (K_2_SO_4_ and KH_2_PO_4_), 57 Ca (CaCl_2_), and 40 Mg (MgSO_4_). The basal micronutrient composition (in mg L^−1^) is 2.0 Fe (Fe-EDTA), 0.2 B (H_3_BO_3_), 0.5 Mn (MnCl_2_·4H_2_O), 0.05 Mo [(NH_4_)6MO_7_O_24_·4H_2_O], 0.01 Zn (ZnSO_4_·7H_2_O), and 0.01 Cu (CuSO_4_·5H_2_O) (Hoagland and Arnon, 1950). Dicyandiamide, a nitrification inhibitor, was added to prevent oxidation of ammonium. The pH and the EC of the nutrient solution used were 6.89 and 1005 μS cm^−1^, respectively. The *P. notoginseng* seedlings were watered with Hoagland nutrient solution every 3 d during the cultivation.

### Fungus strains and growth conditions

A trial strain was isolated from the rotten root of *P. notoginseng* and identified as *F. oxysporum, F. solani*, and *C. destructans* by Sangon Biotech Co., Ltd (Shanghai, China). After activating 4 times on PDA medium, more vigorous strains were generated.

### Sample preparation

Five kinds of traditional Chinese medicinal materials were purchased from Yunnan Jinfa Pharmaceutical Limited Company (Kunming, Yunnan of China). Medicinal parts of *Kaempferia galanga* L., *Zingiber officinale* Roscoe, and *Curcuma Longa* L. were dry rhizoma. *Alpinia oxyphylla* Miq. was the dried ripe fruit. *Alpinia katsumadai* Hayata was dry seed. The EOs from five Zingiberaceae plants species, identified by Yong-Xian Cheng, were prepared by steam distillation for 7 h with 8-fold excess water (v w^−1^). The EOs were collected and dried by using sodium sulfate and then stored at −20°C before being used.

### Oxford cup experiment

A mycelium block was obtained with a 5 mm diameter hole punch and placed in the middle of the Petri dish. The four Oxford cups were then placed at the same distance around the mycelium block, where the distance between an Oxford cup and the middle of the Petri dish was 25 mm. Then, 200 μL of EOs were added to the Oxford cup. A solution of 10/1000 dimethyl sulfoxide (DMSO) and 1/1000 Tween 80 was used as a negative control, and 5 mg mL^−1^ flutriafol was used as a positive control. Each group was prepared in an identical manner four times. Finally, the culture dishes were placed in a microbiological incubator at 28°C. *F. oxysporum* and *F. solani* were cultured for 4 d and *C. destructans* was cultured for 9 d. Radial growth (RG) of the fungi was determined based on the average value of two perpendicular diameters (Pan et al., 2011). The growth inhibition rate was calculated using the following equation:

$$\begin{array}{l} \text{Growthinhibition rate}=\;\frac{\text{RG of negative control}-\text{RG of treated Sample}}{\text{RG of negative control}}\times 100\% \end{array}$$

### IC_50_ experiment

The experiments above led to the identification of EOs from *A. Katsumadai* and *Z. Officinale* as having an inhibition rate of more than 30%. The IC_50_ values of these EOs were determined using the method of Ikematsu et al. (2017). Each EO was dissolved in a solution of 10/1000 DMSO and 1/1000 Tween 80, and then diluted two-fold with the same solution to adjust the concentration to 1.17–600 mg mL^−1^. A mixture formed from the filter-sterilized EOs (20 μL) and a quarter PDA growth medium without agar (150 μL) was added to cells of a 96-cell microtiter plate. The concentrations of conidial suspensions were adjusted to be 1 × 10^6^ spores mL^−1^ for each fungus. Then, a standardized suspension of the fungus (30 μL) was added to each well. The mixture of 150 uL of the PDA medium without agar and 50 μL of 10/1000 DMSO and 1/1000 Tween 80 solution was used as a negative control. Hymexazol was used as a positive control. The plates, securely sealed with a polyester sealing film (VWR), were incubated in a fungal incubator at 28°C for 36 h, and the absorbance of each well was measured at 595 nm by an enzyme-labeled instrument (SkanIt RE 4.1).

### GC-MS analysis and compound identification of EOs from *A. katsumadai* or *Z. officinale*

Chemical compositions of EOs were analyzed using Gas Chromatography-Mass Spectrometry (GC-MS). The GC apparatus used (Agilent Technology-provided equipment model) was equipped with an HP-5MS capillary column (30 m × 0.25 mm, film thickness of 0.25 μm). The oven temperature was initially set at 50°C for 2 min and then raised up to 130°C (at a rate of 5°C min^−1^), subsequently by 4°C min^−1^ up to 190°C, and then by 20°C min^−1^ up to 220°C and held for 5 min. The electron ionization source was set at 70 eV. The detector and injector temperatures were set at 250°C and 230°C, respectively. Helium was used as the carrier gas at a flow rate of 1.0 mL min^−1^. The scanned mass range was 30–550. The constituents of EOs were identified by comparing their retention time and mass spectra with those of the authentic samples contained in the NIST14 (National Institute of Standards and Technology-mass spectral) database to obtain the final assignments (Stein, 2005).

### Antifungal activities of principal components of EOs from *A. katsumadai* or *Z. officinale*

The above analysis showed that eucalyptol is the major component of the of the EOs from *A. katsumadai* and *Z. officinale*, accounting for more than 30%, and the second largest component of *A. katsumadai* is geranyl acetate and that of *Z. officinale* is α-terpineol. Oxford cup experiments were carried out with the three principal components. A solution of eucalyptol and geranyl acetate (W:W = 413.34 mg:186.66 mg) was prepared containing approximately the natural abundance amounts of the EOs in *A. katsumadai*. In the same way, eucalyptol and α-terpineol (W:W = 457.32 mg:142.68 mg) were mixed to obtain a solution of the EOs of *Z. officinale*. Flutriafol and hymexazol were used as positive controls and 10/1000 DMSO and 1/1000 Tween 80 mixture was used as a negative control. Eucalyptol (purity: 99%) was purchased from Shanghai Saen Chemical Technology Limited Company, while geranyl acetate (purity: ≥96%) and α-terpineol (purity: 96%) were purchased from Shanghai Yuanye Biotechnology Limited Company.

### Preparation of petroleum ether extract (pee) from *Z. officinale*

Fresh *Z. officinale* (10.29 kg) was purchased from Shenzhen Vegetable Market and identified by Yong-Xian Cheng. The material was cut into slices and extracted with petroleum ether (60–90°C) under ultrasound at room temperature (3 × 30 L × 1 h). The extract was concentrated under reduced pressure to afford a petroleum ether extract (8.35 g).

### The effect of the pee from *Z. officinale* on the incidence of *P. notoginseng in vivo*

A 1:2 ratio of sterilized quartz sand and roseite was used as the *P. notoginseng* culture medium. Various concentrations of PEE were mixed into the matrix (0 mg g^−1^, 0.2 mg g^−1^, and 0.4 mg g^−1^). Healthy *P. notoginseng* seedlings were submerged in a sterilized or mixed conidial suspension containing 1 × 10^6^ spores mL^−1^ of *F. oxysporum* for 2 h.

Four groups were set up as follows:

Negative Control (NC): healthy plants without the PEE and *F. oxysporum* infection;
Positive Control (PC): healthy plants without the PEE but with *F. oxysporum* infection;
0.2: healthy plants with 0.2 mg g^−1^ of the PEE and *F. oxysporum* infection;
0.4: healthy plants with 0.4 mg g^−1^ of the PEE and *F. oxysporum* infection.

Forty *P. notoginseng* plants were used in each group. After 30 d infection by the pathogen, the plants were graded for severity of wilt disease as 0 (not showing chlorosis), 1 (the stem is soft), 2 (the stem has fallen, but the leaf has not wilted), and 3 (plant wilting), and assigned a Disease index = ∑(rating × number of plants rated)/(total number of plants × highest rating) × 100; and a Disease incidence = (number of infected plants/total number of plants) × 100%.

### Determination of fresh weight of plant

The *P. notoginseng* plants were washed with tap water and then with distilled water, and finally dried with moisture absorbing paper. The whole plant was weighed to obtain the total fresh weight. The plants were divided into two parts from the stem base and each part was weighed.

### Determination of chlorophyll content

After *F. oxysporum* infection for 30 d, the chlorophyll contents of plants were determined by using a SPAD-502 type chlorophyll meter, which gave SPAD values.

### Determination of photosynthetic index

The P_n_, g_s_, C_i_, and T_r_ of new fully expanded leaves of *P. notoginseng*, after *F. oxysporum* infection for 30d, were determined using a portable photosynthesis open system (model 6400; Li-COR, Lincoln, NE). The leaf temperature and relative humidity remained at 28°C and 50%, respectively, and the light flux intensity was 600 μmol photons m^−1^ s^−1^. Data were recorded after equilibration (approximately 10 min) (Dong et al., 2018).

### Determination of the activity of POD and cat

Using procedures developed earlier (Zhang et al., 2017) and also by our research group, we determined the activities of POD and CAT by using kits purchased from Beijing Solarbio Science (China). All measurements were performed according to the manufacturer's instructions.

### Determination of the content of MDA

Leaf pieces (0.2 g) were homogenized in 5 mL of 5% (w v^−1^) trichloroacetic acid, and the homogenates were centrifuged at 3,000 g for 10 min. Portions (2 mL) of the supernatant was mixed with 2 mL of 0.67% (w v^−1^) thiobarbituric acid. The mixtures were incubated in boiling water baths for 10 min, cooled to room temperature, and then centrifuged at 3,000 g for 30 min. The absorbances of the supernatants were measured at 450, 532, and 600 nm (Xie et al., 2015).

### Determination of root activity

Root samples (0.5 g) were mixed thoroughly with a 0.4% TTC (2, 3, 5-Tripheyl Tetrazolium Chloride) solution and transferred to test tubes. The reaction was stopped by adding 2 mL of 1 M H_2_SO_4_ after incubation for 1.5 h at 37°C. The roots were ground with 4 mL ethyl acetate and the volume was increased to 10 mL before the absorbance was measured at 485 nm with ethyl acetate as a blank (Zhang and Chen, 2008) to obtain the Tetrazolium reduction intensity = Tetrazolium reduction (mg)/[Root weight (g)] [Time (h)]

### Inhibitory effects of volatile and non-volatile components of the pee from *Z. Officinale* on *F. Oxysporum*

The EOs (0.90 g) were extracted from the PEE (4.0 g) of *Z. officinale* by steam distillation with 100 mL of distilled water. The residual non-volatile portion was 0.60 g. The EOs and the non-volatile portions were respectively dissolved in solutions of 10/1000 DMSO and 1/1000 Tween 80 to obtain the final concentration of 50 mg mL^−1^. The effects of different portions on pathogens were determined using the Oxford cup method described above.

### Statistical analysis

Statistical analysis was performed with an IBM SPSS Statistics 19.00 using one way ANOVA and Duncan (D) multiple comparisons test.

## Results

### The inhibition effect of EOs from five medicinal materials of zingiberaceae

**Figure 1:**
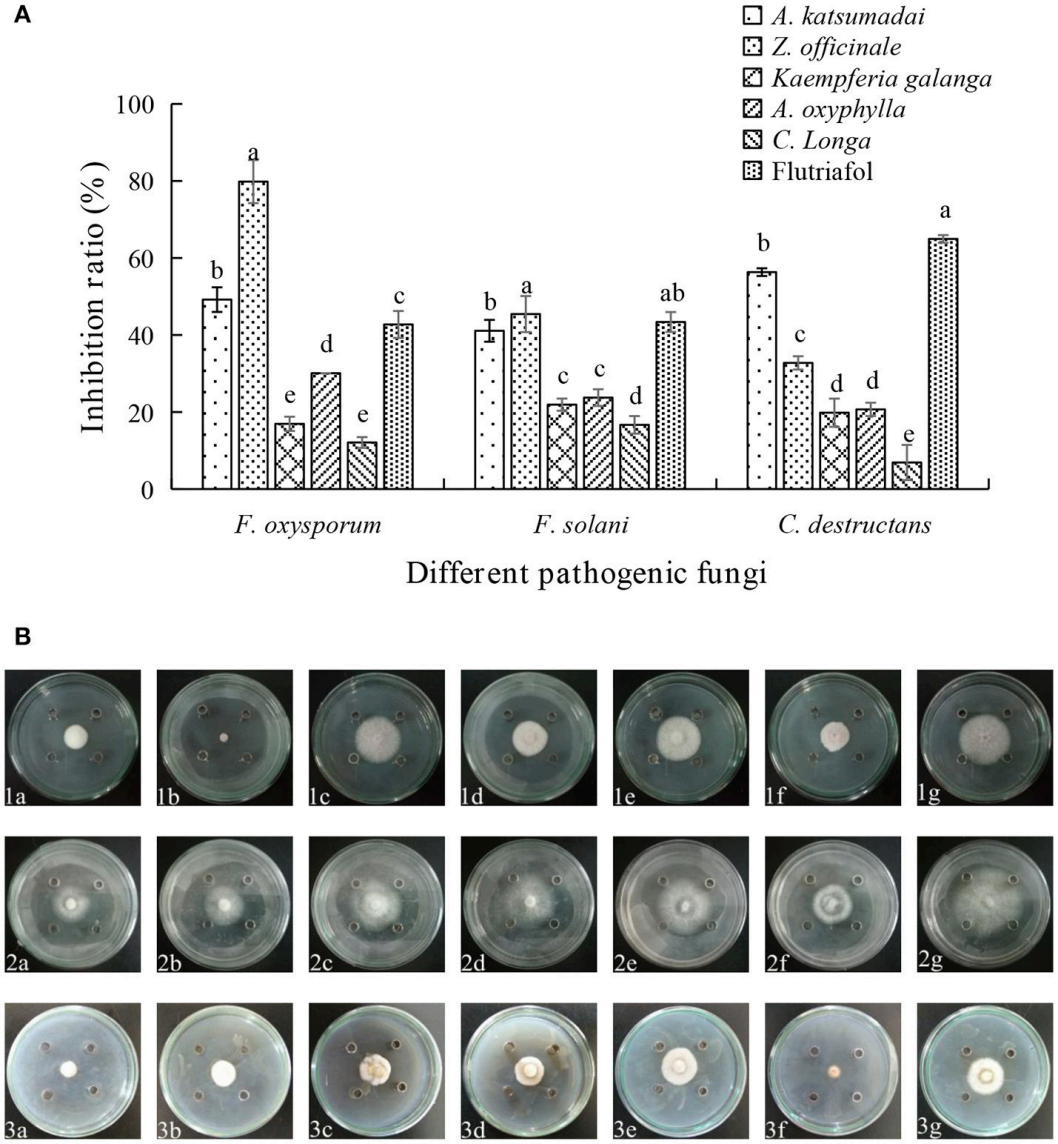
(**A)** Effect of EOs from five Zingiberaceae plants on the growth of three kinds of root-rot fungi of *P. notoginseng*. **(B)** Inhibition of EOs from five Zingiberaceae plants on three species of fungi. The three species of fungi were (1) *F.oxysporum*, (2) *F. solani*, and (3) *C. destructans. Five* Zingiberaceae plants were (a) *A. katsumadai*, (b) *Z. officinale*, (c) *Kaempferia galanga*, (d) *A. oxyphylla*, and (e) *C. longa*; (f) Flutriafol as Positive Control, and (g) 10/1000 DMSO and 1/1000 Tween 80 mixture were the Negative Control. Each data point represents the mean ± SD of five replicates. Different letters represent significant differences (*P* < 0.05) among different treatments.

As shown in Figures 1A,B, five species of Zingiberaceae EOs display different degrees of inhibition against three fungi. The inhibition extents of *A. katsumadai* EO on *F. oxysporum, F. solani*, and *C. destructans* were 49.19, 41.10, and 56.32%, respectively. The inhibition by *A.katsumadai* EO against *C. destructans* was stronger than that by *Z. officinale* EO, whereas the inhibition by *Z. officinale* EO toward *F. oxysporum* was stronger than that by *A. katsumadai* EO. Specifically, the inhibition extent of *Z officinale* EO against *F. oxysporum* could reach 79.83% at a concentration of 50 mg mL^−1^. The results clearly show that the inhibition by EOs from *A. katsumadai* and *Z. officinale* on the three fungi are stronger than those of the EOs derived from the other three plants. Therefore, we chose these two EOs to proceed with the experiment.

### Determination of IC_50_ values

The inhibitory activities against three fungal strains correspond to IC_50_ values ranging from 16.65 to 109.96 mg mL^−1^. As shown in Table 1, the EO from *A. katsumadai* could inhibit *F. oxysporum, F. solani*, and *C. destructans* with IC_50_ values of 21.86 mg mL^−1^, 109.96 mg mL^−1^, and 84.82 mg mL^−1^, respectively. Similar effects are brought about by the EO from *Z. officinale* with an exception to its effect on *F. oxysporum*, which is more sensitive to the EO from *A. katsumadai*.

**Table 1:** IC_50_ Determination of EOs from *A. katsumadai* or *Z. officinale* (mg mL^−1^).

**EOs**	***F. oxysporum***	***F. solani***	***C. destructans***
*A. katsumadai*	21.86 ± 0.39	109.96 ± 1.26	84.82 ± 0.95
*Z. officinale*	79.04 ± 0.39	69.28 ± 3.44	94.34 ± 2.56
Hymexazol	22.09 ± 1.89	28.00 ± 1.29	16.65 ± 0.77

### Analysis of EOs from *A. katsumadai* and *Z. officinale* by GC-MS

**Figure 2:**
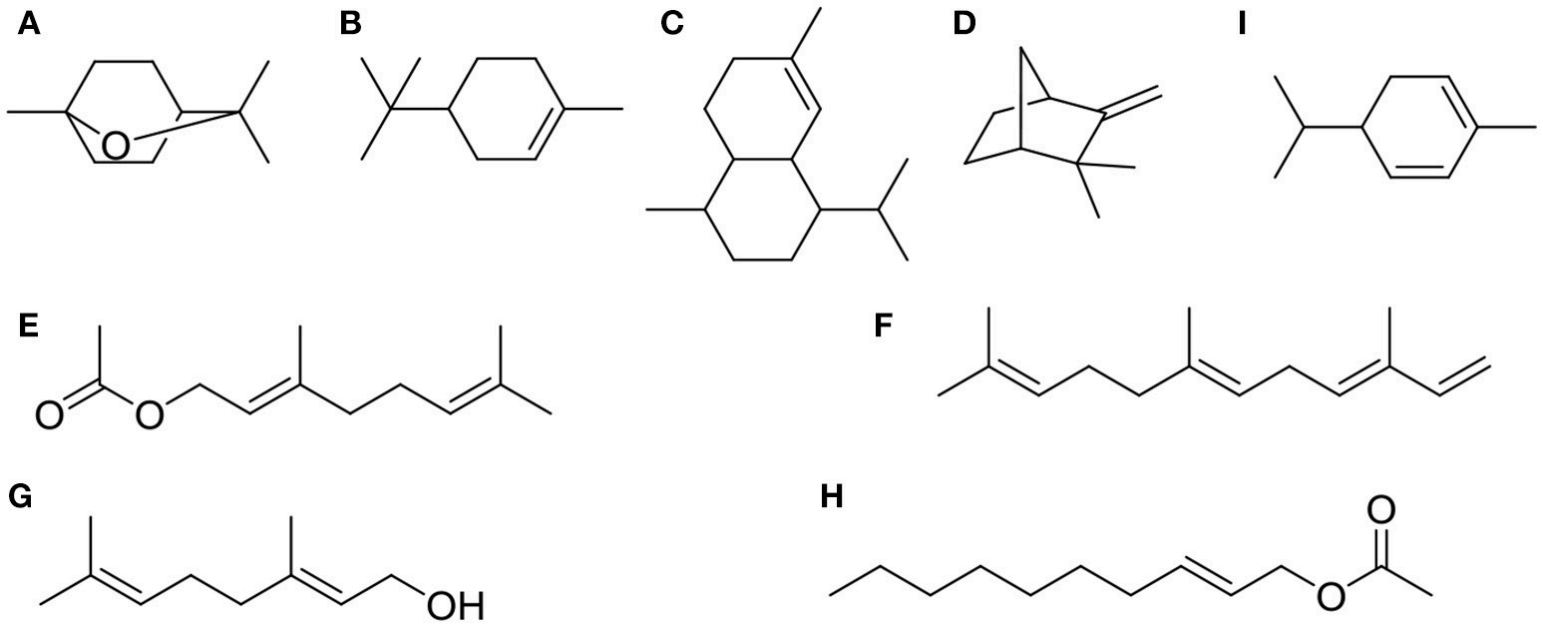
The top five chemical structures of *A. katsumadai* and *Z. officinale* EOs through GC-MS analysis. **(A)** eucalyptol, **(B)** α-terpineol, **(C)** naphthalene,1,2,3,4,4a,5,6,8a-octahydro-7-methyl-4-methylene-1-(1-methylethyl)-,(1α,4aβ,8aα)-(1α,4aβ,8aα)-, **(D)** camphene, **(E)** α-farnesene, **(F)** geranyl acetate, **(G)** geraniol, **(H)** (*E*)-2-decenyl acetate, and **(I)** α-phellandrene.

Chemical compositions of EOs from *A. katsumadai* and *Z. officinale* were determined by using GC-MS. It was found that the top five major compounds in EO of *A. katsumadai* are eucalyptol (30.03%), geranyl acetate (13.56%), geraniol (6.67%), (*E*)-2-decenyl acetate (6.42%), and α-phellandrene (4.84%). The principal compounds in the EO of *Z. officinale* are eucalyptol (35.33%),α-terpineol (11.02%), naphthalene,1,2,3,4,4a,5,6,8a-octahydro-7-methyl-4-methylene-1-(1methylethyl)-,(1α,4aβ,8aα)-(1α,4aβ,8aα)- (5.65%), camphene (4.11%), and α-farnesene (3.47%) (Tables S1, S2). Among these substances, only eucalyptol is present in both plants and it is the most abundant component (Figure 2).

### Antifungal properties of compounds from *A. katsumadai* and *Z. officinale*

**Figure 3:**
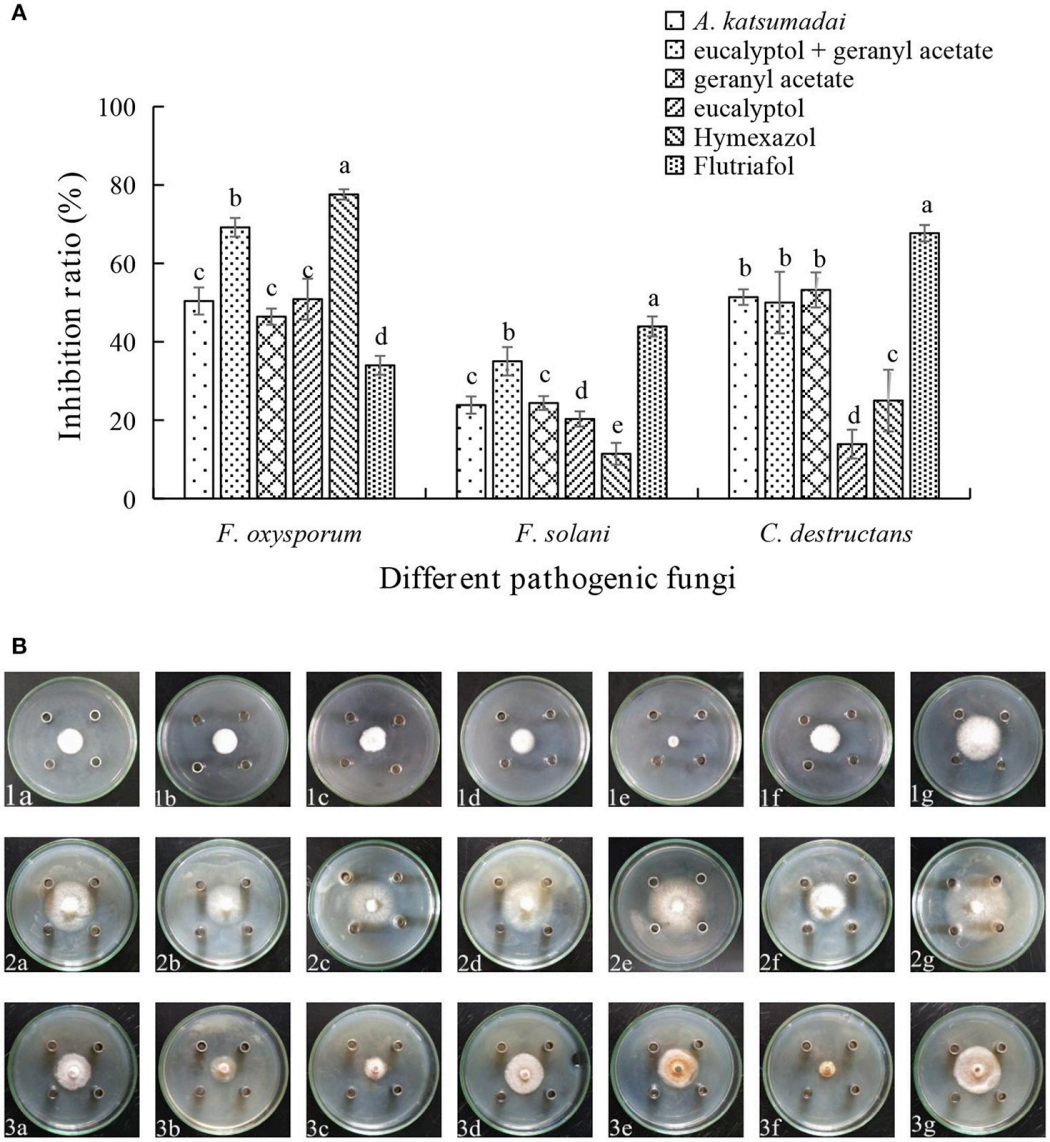
**(A)** Inhibition ratio of principal compounds from *A. katsumadai* EO on *three* fungi. **(B)** Inhibition of principal compounds from *A. katsumadai* EO on three fungi. The three species of fungi were (1) *F.oxysporum*, (2) *F. solani*, and (3) *C. destructans*. Seven treatments were (a) *A. katsumadai* EO, (b) eucalyptol + geranyl acetate, (c) geranyl acetate, (d) eucalyptol, (e) Hymexazol, and (f) Flutriafol was the Positive Control and (g) 10/1000 DMSO and 1/1000 Tween 80 mixture was the Negative Control. Each data point represents the mean ± SD of five replicates. Different letters represent significant differences (*P* < 0.05) among different treatments.

**Figure 4:**
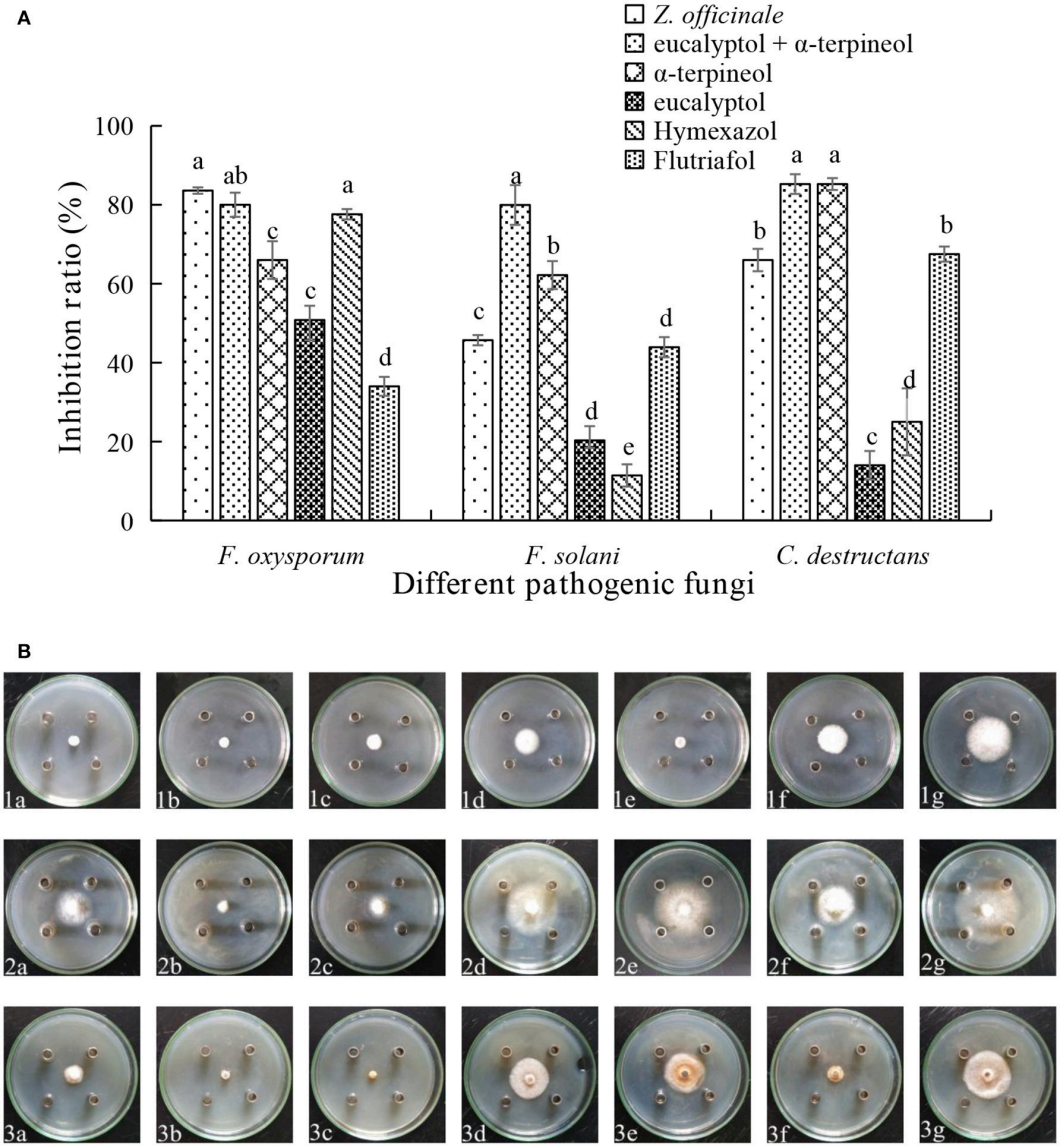
**(A)** Inhibition ratio of principal compounds from *Z.officinale* EO on *three* fungi. **(B)** Inhibition of principal compounds from *Z.officinale* EO on three fungi. The three species of fungi were (1) *F.oxysporum*, (2) *F. solani*, and (3) *C. destructans*. Seven treatments were (a) *Z.officinale* EO, (b) eucalyptol + α-terpineol, (c) α-terpineol, (d) eucalyptol, (e) Hymexazol, and (f) Flutriafol was the Positive Control, and (g) 10/1000 DMSO and 1/1000 Tween 80 mixture was the Negative control. Each data point represents the mean ± SD of five replicates. Different letters represent significant differences (*P* < 0.05) among different treatments.

Assessments of the antifungal activities of the principal components of the EOs showed that geranyl acetate inhibits *F. oxysporum, F. solani*, and *C. destructans* with the latter fungus being the most sensitive (53.24% inhibition). Eucalyptol is also active toward these fungi strains, but in this case, it appeared that *F. oxysporum* is the most sensitive with an inhibition rate of 50.88%. Interestingly, synergistic effects were observed to exist between eucalyptol and geranyl acetate, a mixture of which exhibits strong growth inhibition toward *F. oxysporum, F.solani*, and *C. destructans* with inhibition of 69.20, 35.03, and 50.00%, respectively (Figures 3A,B). By comparing the potency of α-terpineol and a mixture of it with eucalyptol, it was found that it displays comparable antifungal effects, thereby suggesting that all the three fungi strains are sensitive to α-terpineol with inhibition effects being 66.00, 62.18, and 85.25%, respectively. Finally, the antifungal potencies of α-terpineol against *F.solani* and *C. destructans* are much higher than that of the EO of *Z. officinale*. This observation suggests that α-terpineol is one of the major active components responsible for the antifungal properties of the EO of *Z. officinale*. Also, we found that this EO displays much stronger inhibition of *F. oxysporum* than does α-terpineol (Figures 4A,B).

### *In vivo* effects of the petroleum ether extract (pee) of *Z. officinale*

**Figure 5:**
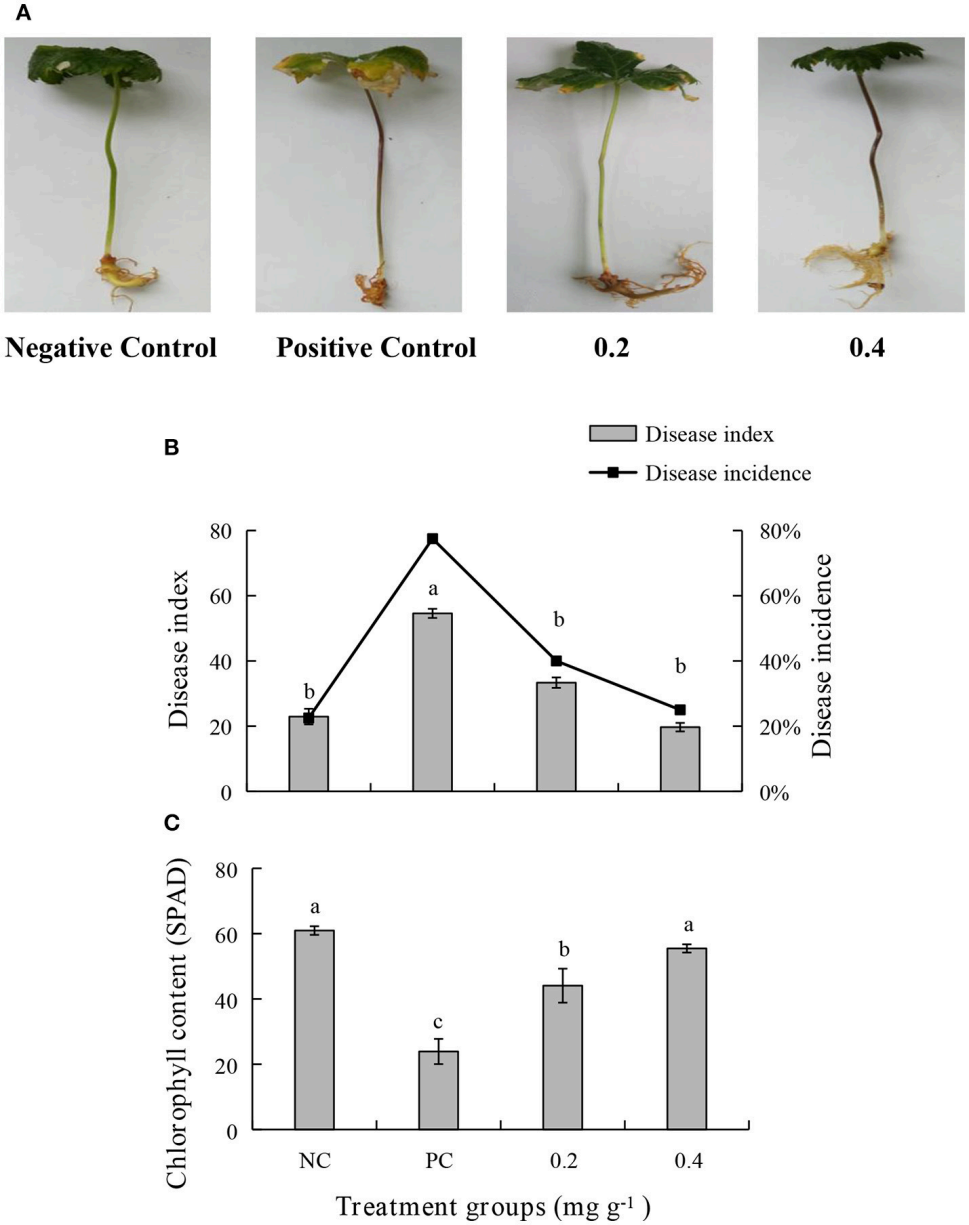
Effects of different treatments on **(A)** symptoms of disease, **(B)** disease index, disease incidence, and **(C)** chlorophyll content of *P. notoginseng*. Plant disease index in different treatment groups. Negative Control (NC): healthy plants without Petroleum Ether Extract (PEE) and *F. oxysporum* infection; Positive Control (PC): healthy plants without PEE but with *F. oxysporum* infection; 0.2: plants with 0.2 mg g^−1^ PEE and *F. oxysporum* infection; 0.4: plants with 0.4 mg g^−1^ PEE and *F. oxysporum* infection. Each data point represents the mean ± SD of five replicates. Different letters represent significant differences (*P* < 0.05) among different treatments.

An *in vivo* experiment was conducted by using PEE to simulate the effects of the EOs. The growth of plants treated with 0.4 mg g^−1^ of PEE is significantly higher than those treated with 0.2 mg g^−1^ of PEE and the positive control (PC), and is similar to that of the negative control (NC) (Figure 5A). The fresh weight of the above-ground parts, under-ground parts, and the total fresh weight of 0.4 mg g^−1^ PEE treated plants are, respectively, 52.26, 28.78, and 44.20%, which are higher than the PC (**Figure 7A**). The results show that the disease index and incidence of *P. notoginseng* is reduced by treatment with PEE after *F. oxysporum* infection (Figure 5B). Upon infection of the pathogenic fungi, the chlorophyll content of the plants gradually decreases. When the amount of PEE added is 0.4 mg g^−1^, the content of chlorophyll is 2.3 times higher than that of the PC and close to the NC (Figure 5C).

**Figure 6:**
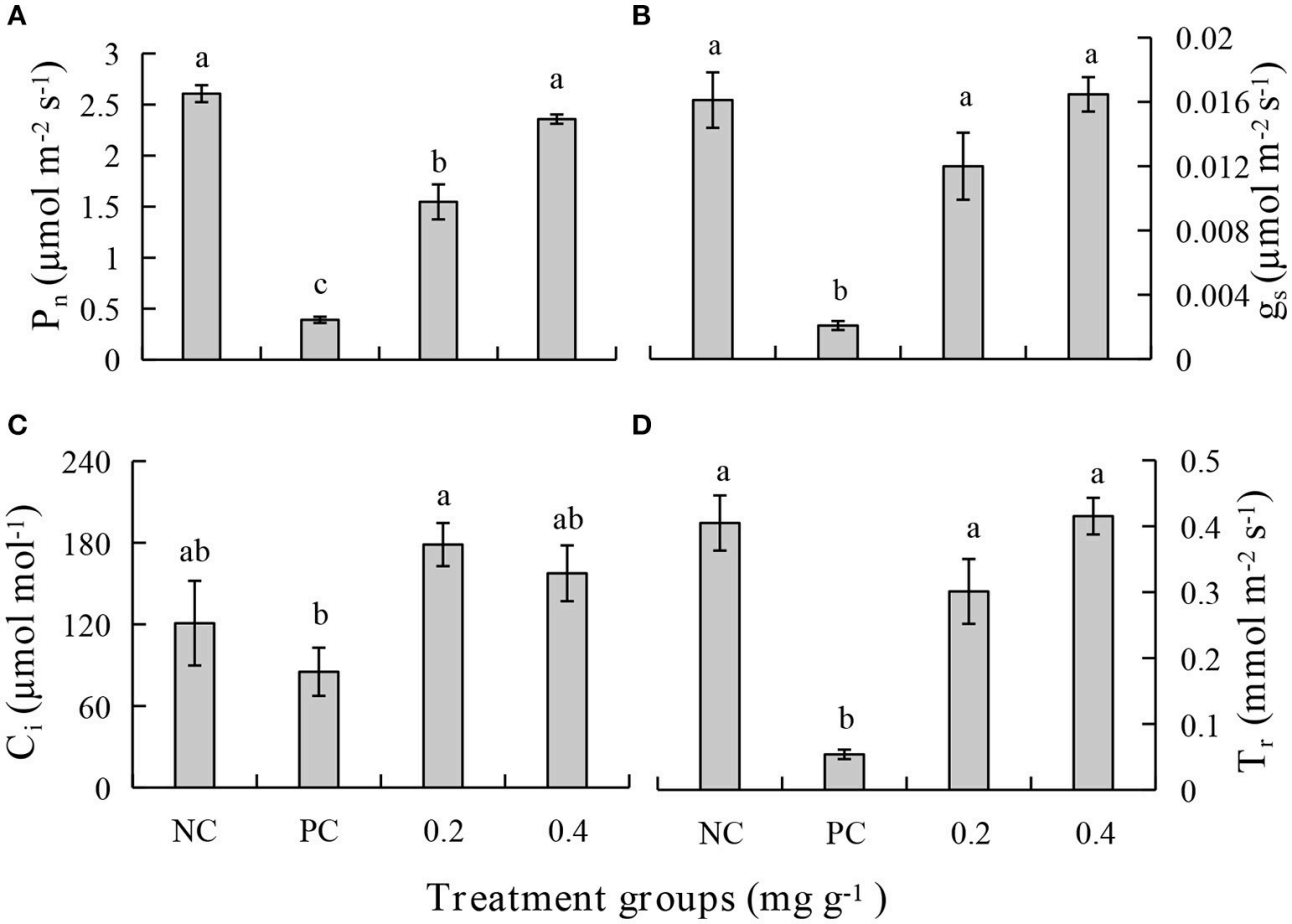
Effects of different treatments on **(A)** photosynthetic rate (P_n_), **(B)** stomatal conductance (g_s_), **(C)** intercellular CO_2_ concentration (C_i_), and **(D)** transpiration rate (T_r_) of *P. notoginseng*.

After treatment with PEE, the P_n_, g_s_, C_i_, and T_r_ were increased significantly and are higher in the 0.4 mg g^−1^ PEE treated plant when compared to the PC. Compared with the PC, the extents of increase of P_n_, g_s_, C_i_, and T_r_ in 0.4 mg g^−1^ PEE treated plants are 83.47, 87.50, 45.96, and 88.10%, respectively (Figure 6).

**Figure 7:**
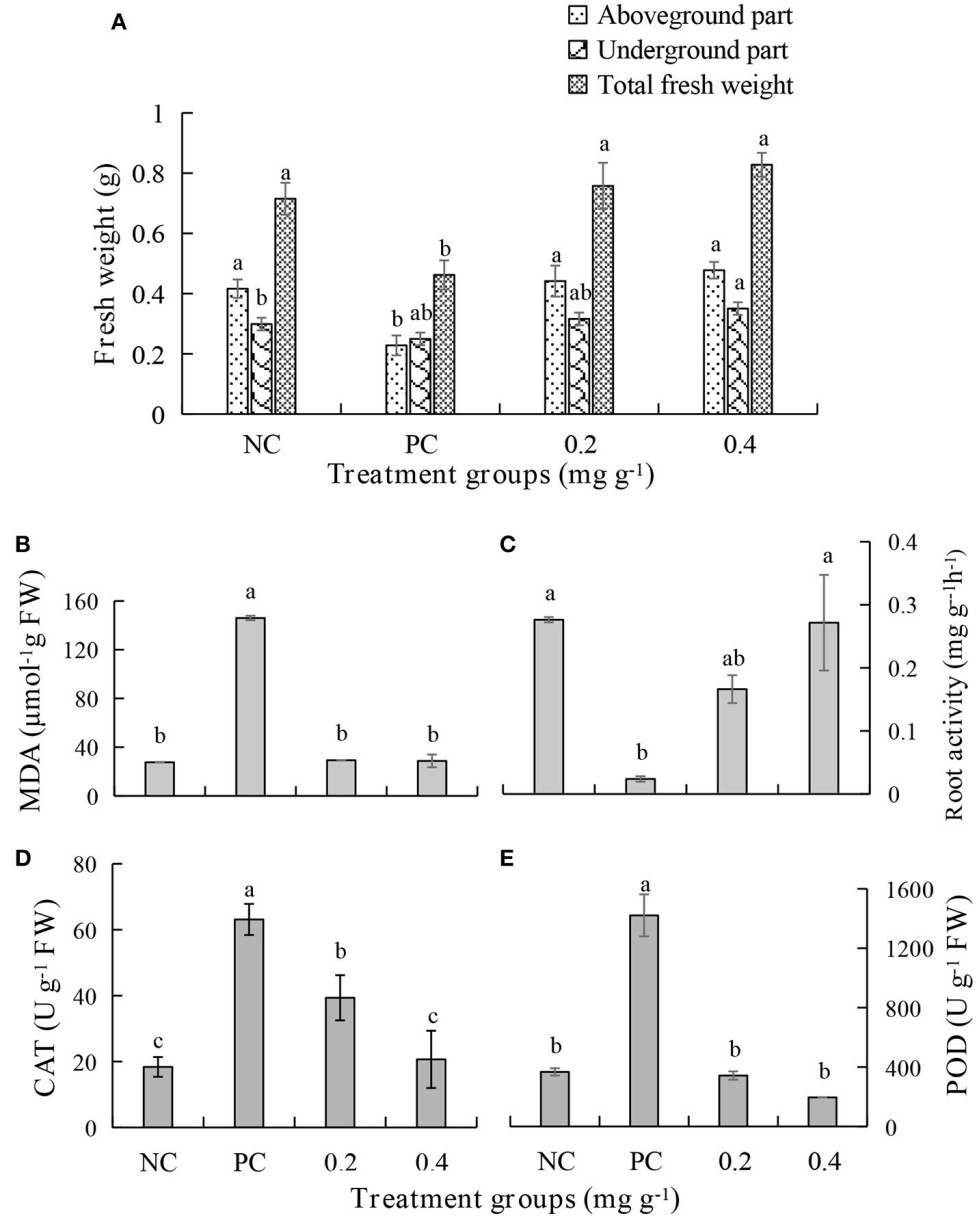
Effects of different treatments on **(A)** fresh weight, **(B)** the malondialdehyde (MDA) content, **(C)** root activity, **(D)** the activities of catalase (CAT), and **(E)** peroxidase (POD) of *P. notoginseng*.

The malondialdehyde (MDA) content increases when the plant is diseased. Treatment with PEE at the concentration of 0.2 mg g^−1^ or 0.4 mg g^−1^, causes a 5-fold decrease in the content of MDA, which is equivalent to the level in a healthy plant (Figure 7B). The root activity of the plant significantly decreases following pathogen infection but increases after treatment with PEE. Specifically, the root activity increases 7.0- or 11.4-fold when the plants are treated with 0.2 mg g^−1^ or 0.4 mg g^−1^ PEE, which is close to the value of the NC (Figure 7C). In addition, the activities of the enzymes catalase (CAT) and peroxidase (POD), which relate to the infection by pathogenic fungi, were determined. The results show that the activities of the two enzymes increase with the severity of the disease. The CAT activity of plants treated with PEE at doses of 0.2 mg g^−1^ and 0.4 mg g^−1^ was respectively reduced by 1.6 and 3.1 times compared with the PC, and the 0.4 mg g^−1^ treatment level was found to be close to that of the NC (Figure 7D). Moreover, PEE causes a reduction of POD activity in a dose-dependent manner. It was observed that a 4.1- or 7.3-fold reduction of the POD activity is promoted by treatment with 0.2 mg g^−1^ or 0.4 mg g^−1^ of PEE, respectively (Figure 7E).

### Inhibition of volatile and non-volatile components of pee on *F. oxysporum*

To determine if the effects of the EOs are equivalent to those of PEE, an experiment was carried out to compare the antifungal potency of the volatile and non-volatile portions of the PEE. The results show that the volatile portion inhibits *F. oxysporum* with an inhibition extent of 70.97% but the non-volatile portion promotes only 3.23% inhibition, thereby supporting the rationale of using PEE as a substitute for EOs (Table 2, Figure S1).

**Table 2:** Inhibitory effects of volatile or non-volatile portion of PEE from *Z. officinale* on *F. oxysporum*.

**Different treatment**	***F. oxysporum***	
	**Colony diameter (mm)**	**Inhibition ratio (%)**
Volatile portion	9.00 ± 9.44^c^	70.97 ± 3.95^a^
Non-volatile portion	30.50 ± 1.08^a^	3.23 ± 1.62^c^
Flutriafol	18.88 ± 0.48^b^	39.11 ± 1.54^b^
Negative control	31.00 ± 0.82^a^	0.00 ± 1.86^c^

*Different letters in the same column represent significant differences (P < 0.05) among different treatments*.

## Discussion

In recent years and with the increased economic value of *P. notoginseng*, questions have been raised about the methods used to plant *P. notoginseng*. Problems related to diseases caused by intensive planting are becoming more serious, especially those associated with root rot. At present, many methods exist for controlling root-rot disease, such as chemical, physical, and biological treatments. The most commonly used control method is soil fumigation with a synthetic chemical. However, pesticides are banned in organic agriculture because the residues of these substances in foods have long-term health effects (Akoto et al., 2013). In addition, pesticides accumulate in the soil and exert adverse effects on the beneficial soil microflora (Ahemad and Khan, 2011).

The antibacterial mechanism of action of plant EOs involves several events including the destruction of cell membranes, exudation of cell contents, condensation of cytoplasm, and change of membrane osmotic pressure (Gustafson et al., 1998; Lambert et al., 2001; Devi et al., 2010). At present, few reports exist describing the bacteriostatic mechanism of the EOs of Zingiberaceae, but some contain the suggestion that lipophilic substances in the EOs of *Z. officinale* play a more important role in the bacteriostatic process. These substances can penetrate the fungi cell membrane, react with the enzymes on the membrane, destroy the enzymatic system of fungi, and further damage the function of their genetic material. In addition, the EOs can react with the proteins on the cell membranes, destroy phospholipid bimolecular layers and cell structures, make more EOs infiltrate into the cells, and eventually lead to the death of fungi (Farag et al., 1989; Sikkema et al., 1994; Abd EI-Baky and Baroty, 2008; Elbaroty et al., 2010).

The aim of the current study was to assess the use of environment-friendly EOs from five medicinal plants in the Zingiberaceae family by determining their inhibitory effects on the growth of three main soil-borne pathogens associated with the root-rot disease of *P. notoginseng*. The findings reveal that the EOs from *A. katsumadai* or *Z. officinale* significantly reduce mycelium growth of the test pathogens *in vitro* (Figures 1A,B). This result is similar to the previously reported inhibitory effect of *Z. officinale* on *F. oxysporum* (Ginting et al., 2013). The EO of *A. katsumadai* has a similar or lower IC_50_ value in comparison to hymexazol toward the three tested fungi. The *A. katsumadai* EO was found to be the most effective for inhibition of *F. oxysporum* with an IC_50_ value of 21.86 mg mL^−1^ (Table 1). In addition, it has been reported that *Amomum tsao-ko* also displays an inhibitory effect on *F. oxysporum* (Sun et al., 2018).

GC-MS was employed to elucidate the chemical substances responsible for the antifungal properties of the EOs. In this study, 54 and 60 compounds were identified as being present in the EOs from *A. katsumadai* and *Z. officinale*, respectively (Tables S1, S2). The findings show that eucalyptol is present in both plants as the principal component, and that α-terpineol is the second major component. It was previously reported that the principal components of EO extracted from plants, among which eucalyptol is present to the extent of 30%, also have inhibitory effects on some fungi (Marei et al., 2012). In order to further determine if the abundant compounds of EOs are active, eucalyptol and α-terpineol were tested *in vitro* individually and as a mixture. It was found that the antifungal activities of mixtures of the principal compounds are greater than those of the individual compounds but less than the parent EO (Figures 3A,B, 4A,B). Studies have shown that eucalyptus displays different degrees of inhibition against the four plant pathogenic fungi, *Rhizoctonia solani, Fusarium oxysporum, Penecillium digitatum*, and *Asperigallus niger* (Marei et al., 2012).

It had been reported that the EOs not only affect the normal functions of the cell membranes but they also perturb the stability of the lipid layer of the cell membranes. In earlier studies, eucalyptol and α-terpineol were identified as oxygenated monoterpenes components of *Z. officinale* (Kordali et al., 2016). The mechanisms of antifungal action of monoterpenes such as camphene, (*R*)-camphor, (*R*)-carvone, 1,8-cineole, and cuminaldehyde were not fully elucidated. However, the results of several studies led to the conclusion that these substances inhibit pectin methyl esterase, thereby promoting changes in the degrees of methyl esterification of pectins, which are major components of the fungi cell walls (Marei et al., 2012). It was speculated that PG enzymes might play an important role in the penetration of the plant root epidermis by *F.oxysporum* and upward expansion of the xylem (Beckman, 1987).

According to the GC-MS analysis carried out by Cai et al. (2008), the components and their relative amounts of the PEE from *Anoectochilus roxburghii* (Wall.) Lindl. are almost the same as those of its EOs. The method of EO and PEE extraction employed by us are the same as that used by Cai. A total of 72 components were characterized, accounting for 97.70% of the Eos, and69 components were identified, accounting for 95.40% of PEE. Moreover, the main components in both the EOs and PEE are aliphatic compounds. Also, the PEE extraction process is relatively simple and the yield is higher. Owing to this, we have conducted *in vivo* experiments using PEE instead of EO, which were aimed at determining the physiological indexes of *P. notoginseng*. The results show that the occurrence and severity of *P. notoginseng* root-rot disease is greatly decreased by adding PEE to the culture matrix (Figures 5–7).

In the current study, we observed that degradation of chlorophyll content after infection of *P. notoginseng* by *F. oxysporum* that finally leads to symptoms being displayed by the plant. The leaves of the above-ground part of the plant begin yellowing, and the whole plant wilts when the disease becomes more severe (Figure 5A). These observations are consistent with the previous conclusion that *F.oxysporum* infects the roots, stems, veins, and leaves through the xylem.

The bottom leaves of the host become chlorotic. This change then gradually reaches the top leaves, and finally the whole plant turns yellow, wilts, and then dies (Liang et al., 2014). During the infection process, some pathogenic toxins are secreted and these substances cause wilting and lodging of the *P. notoginseng* plants (Zhao et al., 2017). *F. oxysporum* infection causes the fresh weight of the entire above-ground and under-ground parts of the *P. notoginseng* plants to decrease significantly (Figure 7A). The findings are consistent with the previous results, which showed that the fresh weight of leaves, stems, and roots of *P. notoginseng* decrease significantly after *F. oxysporum* infection (Dong et al., 2018). The disease incidence of *P. notoginseng* plants, in the absence of PEE addition, is up to 77.5% (Figure 5B).

The results of the related studies have shown that *F. oxysporum* infection can significantly reduce photosynthesis occurring in *P. notoginseng* plants (Dong et al., 2018). Our study showed that P_n_, g_s_, C_i_, and T_r_ in the PC treatment are significantly lower than those in the NC treatment (Figure 6). The decrease in P_n_ in infected leaves is a result of stomatal closure or disruption of metabolic pathways of photosynthetic products promoted by water stress caused by disease (Duniway and Slatyer, 1971; Lorenzini et al., 1997; Pinto et al., 2000). It is known that planting of xylem increases the resistance of the plant to water, which leads to water deficit in leaves, thereby decreasing photosynthesis and transpiration of leaves (Bowden et al., 1990; Lorenzini et al., 1997). It was reported that vascular wilt may be caused by disruption to photosynthesis, thylakoid electron transport, carbon reduction cycle, and CO_2_ supply (Allen et al., 1998). In the current study, we found that the reduction of photosynthesis in plants infected with *F. oxysporum* is significantly alleviated by treatment with the PEE from *Z. officinale* (Figure 6). Photosynthesis provides carbohydrates for the growth of plants, and on being infected with *F. oxysporum*, the fresh weight of plants decreased significantly (Figure 7A). Malic acid and hydrogen peroxide are important intermediates in photorespiration (Wingler et al., 2000). Photorespiration is closely related to photosynthetic metabolism (Wingler et al., 1999) and plays an important role in biotic and abiotic stress. However, the relationship between the role of these intermediates in metabolism and disease resistance is unclear. Therefore, it is of great significance to clarify the function of photorespiration in the defense response of *P. notoginseng* against pathogen infection.

Vascular wilt disease is a factor involved in pathogenic fungi and host defense response (Dan, 1990). For example, mycelium, toxin, and host defense responses caused by pathogenic fungi can block the plant vascular bundle tissue, thus reducing the water transport capacity and photosynthetic rate of the plant (Pivonia et al., 2002). However, the issue of plant water stress induced by wilt is still controversial (Lorenzini et al., 1997) and the physiological mechanism of the decrease in photosynthesis induced by wilting is not clear (Nogués et al., 2002). When the plant is infected by a pathogen, the disease or susceptibility of a plant to a disease depends on whether the plant can prevent growth and reproduction of the pathogen. The increased activity of resistance-related enzymes such as phenylalanine ammonia lyase (PAL) and polyphenol oxidase (PPO) are related to plant resistance (Dempsey and Klessig, 1995). It was found earlier that the POD and PPO activities increase significantly with the development of disease in the leaves of plants. Also, the deposition of phenols is an important defense mechanism for fighting pathogen infection, because it plays an important role in hypersensitivity and cell wall enhancement (Franke et al., 1998). Phenols are precursors of lignin and the synthesis of phytoprotectants (Yingsanga et al., 2008). Lignin is a widely distributed polymer, which enhances the ability of plants to resist degradation of pathogen enzymes and plays an important role in the defense response of vascular plants (Huang and Hartman, 1998). Thus, the increased activity of these three enzymes is closely related to cell injury, wound repair, and disease resistance (Préstamo and Manzano, 1993). In addition, root activity helps the plant roots avoiding the absorption of arsenic and other toxic substances to provide protection (Singh et al., 2007). In our studies, the root activity of the PC was found to be significantly lower than that of the NC (Figure 7C). The results of other studies indicate that the increase of root activity is related to the enhancement in the oxidation ability of POD (Tiwari et al., 2002). Also, studies have shown that POD, CAT, and SOD together comprise an antioxidant defense system *in vivo* (Chen, 2016). Importantly, we found that the levels of POD and CAT in the PC are significantly higher than in the NC (Figures 7D,E). MDA is a product of unsaturated lipid peroxidation in biofilms (Ciniglia et al., 2015) and, as a result, its quantity can directly reflect the degree of membrane lipid peroxidation (Draper and Hadley, 1990) and the amount of its accumulation determines the degree of damage to plants (Chowhan et al., 2013). In this light, we observed that after *F. oxysporum* infection the amount of MDA in the PC is significantly higher than in the NC (Figure 7B).

In summary, the findings arising in this study indicate that the EOs from Zingiberaceae or the volatile components of PEE have deleterious effects on *P. notoginseng* root rot. This observation suggests a possible alternative non-chemical pesticide approach for the continuous cropping *of P. notoginseng*. Last but not least, this study should pave the way for the use of Zingiberaceae EOs as effective ingredients during soilless production of *P. notoginseng*, which suppress pathogenic *P. notoginseng-*borne fungi.

## Author contributions

Y-XC and XD designed the experiment, analyzed the data and wrote the paper. Y-JY, C-JC, K-ML, Y-NM, and W-MS performed the experiments. S-WG and F-RX commented on the manuscript. All authors have read and approved the manuscript.

### Conflict of interest statement

The authors declare that the research was conducted in the absence of any commercial or financial relationships that could be construed as a potential conflict of interest.
