# Supplementary material for: The Fight Against Panax notoginseng Root-Rot Disease Using Zingiberaceae Essential Oils as Potential Weapons
Source: Front Plant Sci. 2018 Oct 4;9:1346. doi: 10.3389/fpls.2018.01346 (PMC6180181; doi:10.3389/fpls.2018.01346)
Supplement: Supplementary file 1 [file Table_1.pdf]

SUPPORTING INFORMATION

TABLE S1 | Analysis of chemical composition of EO from *A. katsumadai* by GC-MS

NO.	Compound	Retention time	Content (%)
1	hexanal	3.87	0.01
2	1-hexanol	5.29	0.03
3	heptanal	6.08	0.02
4	bicyclo[3.1.0]hex-2-ene, 2-methyl-5-(1-methylethyl)-	6.76	0.13
5	*α*-pinene	6.94	2.38
6	camphene	7.34	0.07
7	bicyclo[3.1.0]hexane, 4-methylene-1-(1-methylethyl)	8.04	0.22
8	bicyclo[3.1.1]heptane, 6,6-dimethyl-2-methylene-, (1*S*)-	8.12	3.52
9	6-methyl-5-hepten-2-one	8.43	0.03
10	*β*-myrcene	8.54	0.65
11	*α*-phellandrene	8.92	4.84
12	3-carene	9.09	0.13
13	acetic acid hexyl ester	9.19	0.09
14	o-cymene	9.51	0.53
15	*D*-limonene	9.64	2.26
16	eucalyptol	9.72	30.03
17	(3*E*)-3,7-dimethyl-1,3,6-octatriene	9.90	0.07
18	2-pyrrolidinone, 1-methyl-	9.95	0.08
19	*γ*-terpinene	10.51	0.49
20	(*E*)-oct-2-en-1-ol	10.78	0.74
21	cyclooctyl alcohol	10.78	0.74
22	octan-1-ol	10.85	0.25
23	*p*-cresol	11.02	0.03
24	1-methyl-4-(1-methylethylidene)-cyclohexene	11.38	0.23
25	linalool	11.72	0.78
26	1-methyl-4-(1-methylethyl)-2-cyclohexen-1-ol	12.35	0.05
27	(+)-bornan-2-one	13.04	0.05
28	pinocarvone	13.57	0.02
29	2-(4-methylidenecyclohexyl)propan-2-ol	13.69	0.34
30	(-)-1-Isopropyl-4-methyl-3-cyclohexen-1-ol	13.99	0.68
31	*α*-terpineol	14.38	3.51
32	(-)-myrtenol	14.54	0.08
33	farnesol	15.42	0.06
34	2,6-octadienal, 3,7-dimethyl-, (*Z*)-	15.78	0.10
35	geraniol	16.17	6.67
36	3-heptylacrolein	16.34	0.44
37	*trans*-2-dodecen-1-ol	16.55	2.80
38	2-iso-propylbenzaldehyde	17.57	0.58
39	2-Phenyl-2-butenal	18.54	0.46

40	terpinyl acetate	18.77	0.22
41	6-octen-1-ol, 3,7-dimethyl-, acetate	18.85	0.04
42	2-propenal, 2-methyl-3-phenyl-	19.44	0.42
43	geranyl acetate	19.77	13.56
44	dec-2-enyl acetate	20.52	6.42
45	bicyclo[7.2.0]undec-4-ene, 4,11,11-trimethyl-8-methylene-	20.92	0.19
46	2-dodecenal	22.37	0.12
47	*trans*-2-dodecen-1-ol	22.54	0.28
48	(-)-(7*S*)-germacrene *D*	22.98	0.12
49	naphthalene, decahydro-4a-methyl-1-methylene-7-(1-methylethenyl)-, [4aR-(4aα,7α,8aβ)]-	23.16	0.35
50	(*S*)-β-bisabolene	23.93	0.44
51	naphthalene, 1,2,3,4,4a,5,6,8a-octahydro-7-methyl-4-methylene-1-(1-methylethyl)-,(1α,4aβ,8aα)-	24.15	0.12
52	4,7-dimethyl-1-propan-2-yl-1,2,3,5,6,8a-hexahydronaphthalene	24.49	0.26
53	hedycaryol	25.45	0.12
54	nerolidol	26.00	1.18
