# Supplementary material for: The Fight Against Panax notoginseng Root-Rot Disease Using Zingiberaceae Essential Oils as Potential Weapons
Source: Front Plant Sci. 2018 Oct 4;9:1346. doi: 10.3389/fpls.2018.01346 (PMC6180181; doi:10.3389/fpls.2018.01346)
Supplement: Supplementary file 2 [file Table_2.pdf]

**TABLE S2 |** Analysis of chemical composition of EOs from *Z. officinale* by GC-MS

| NO. | Compound | Retention time | Content (%) |
|---|---|---|---|
| 1 | 3-furaldehyde | 4.53 | 0.01 |
| 2 | propanoic acid, 2-methyl-, 2-methylpropyl ester | 6.42 | 0.25 |
| 3 | tricyclo[2.2.1.02,6]heptane, 1,7,7-trimethyl- | 6.62 | 0.08 |
| 4 | *α*-pinene | 6.94 | 3.03 |
| 5 | camphene | 7.34 | 4.11 |
| 6 | propanoic acid, 2-methyl-, 2-methylpropyl ester | 7.54 | 0.02 |
| 7 | bicyclo[3.1.1]heptane, 6,6-dimethyl-2-methylene-, (1*S*)- | 8.12 | 1.62 |
| 8 | sulcatone | 8.42 | 0.10 |
| 9 | *β*-myrcene | 8.54 | 0.38 |
| 10 | 2-methylpropyl 2-methylbutanoate | 8.88 | 0.18 |
| 11 | *α*-phellandrene | 8.91 | 0.09 |
| 12 | isobutyl isovalerate | 8.98 | 0.05 |
| 13 | propanoic acid, 2-methyl-, 3-methylbutyl ester | 9.17 | 0.08 |
| 14 | *D*-limonene | 9.64 | 3.21 |
| 15 | eucalyptol | 9.72 | 35.33 |
| 16 | 2-pyrrolidinone, 1-methyl- | 9.95 | 0.08 |
| 17 | (*E*)-*β*-ocimene | 10.20 | 0.06 |
| 18 | propanoic acid, 2-methyl-, 3-methylbutyl ester | 10.43 | 0.01 |
| 19 | *γ*-terpinene | 10.51 | 0.57 |
| 20 | terpinolene | 11.38 | 0.56 |
| 21 | linalool | 11.72 | 0.30 |
| 22 | butanoic acid, 2-methyl-, 2-methylbutyl ester | 11.85 | 0.11 |
| 23 | butanoic acid, 3-methyl-, 2-methylbutyl ester | 11.97 | 0.05 |
| 24 | fenchol | 12.12 | 0.50 |
| 25 | 1-methyl-4-propan-2-ylcyclohex-3-en-1-ol | 12.74 | 0.03 |
| 26 | (+)-bornan-2-one | 13.03 | 2.68 |
| 27 | *DL*-isoborneol | 13.39 | 0.24 |
| 28 | bornyl chloride | 13.58 | 0.39 |
| 29 | borneol | 13.65 | 1.23 |
| 30 | terpinen-4-ol | 13.99 | 2.24 |
| 31 | *α*-terpineol | 14.39 | 11.02 |
| 32 | cyclohexanol, 1-methyl-4-(1-methylethylidene)- | 14.57 | 0.19 |
| 33 | (1*S*)-2exo-acetoxy-1.3.3-trimethyl-norbornan | 15.21 | 0.38 |
| 34 | 4-phenyl-2-butanone | 15.86 | 0.19 |
| 35 | bicyclo[2.2.1]heptan-2-ol, 1,7,7-trimethyl-,acetate, (1*S*-endo)- | 17.05 | 0.29 |
| 36 | thymol | 17.20 | 0.06 |
| 37 | benzoic acid, 2-methylpropyl ester | 18.17 | 0.18 |
| 38 | eugenol | 18.99 | 0.10 |
| 39 | (+)-cyclosativene | 19.33 | 0.08 |

| | | | |
|---|---|---|---|
| 40 | 8-isopropyl-1,3-dimethyltricyclo[4.4.0.02,7]dec-3-ene | 19.44 | 0.34 |
| 41 | 2-Isopropenyl-5-methylhex-4-enylacetat | 19.74 | 0.34 |
| 42 | phenylethyl butyrate | 20.11 | 0.53 |
| 43 | bicyclo[7.2.0]undec-4-ene, 4,11,11-trimethyl-8-methylene- | 20.53 | 2.23 |
| 44 | *α*-guaiene | 21.52 | 0.50 |
| 45 | (1R,3a*S*,8a*S*)-7-isopropyl-1,4-dimethyl-1,2,3,3a,6, 8a-hexahydro-azulene | 21.67 | 0.08 |
| 46 | (1*E*,4*E*,8*E*)-*α*-humulene | 22.03 | 0.90 |
| 47 | (-)-*β*-santalene | 22.23 | 0.14 |
| 48 | naphthalene, 1,2,3,4,4a,5,6,8a-octahydro-7-methyl-4-methylene -1-(1-methylethyl)-,(1*α*,4a*β*,8a*α*)- | 22.80 | 0.98 |
| 49 | (-)-*α*-muurolene | 22.93 | 0.24 |
| 50 | *α*-farnesene | 23.91 | 3.47 |
| 51 | naphthalene, 1,2,3,4,4a,5,6,8a-octahydro-7-methyl-4-methylene -1-(1-methylethyl)-,(1*α*,4a*β*,8a*α*)-(1*α*,4a*β*,8a*α*)- | 24.16 | 5.65 |
| 52 | naphthalene,1,2,3,5,6,8a-hexahydro-4,7-dimethyl-1-(1-methylethyl)-, (1S-*cis*)- | 24.49 | 1.22 |
| 53 | *cis*-bisabolene | 24.80 | 0.15 |
| 54 | (+)-valencene | 24.92 | 0.49 |
| 55 | selina-3,7(11)-diene | 25.17 | 0.66 |
| 56 | *α*-calacorene | 25.22 | 0.89 |
| 57 | (1*E*,4*E*)-germacrene *B* | 25.30 | 0.24 |
| 58 | selina-3,7(11)-diene | 26.93 | 0.26 |
| 59 | (1*S*,4*R*,4a*S*,8a*R*)-4,7-dimethyl-1-propan-2-yl-2,3,4, 5,6,8a-hexahydro-1H-naphthalen-4a-ol | 28.05 | 0.49 |
| 60 | bergamotol | 32.11 | 0.32 |
