# Supplementary material for: The Fight Against Panax notoginseng Root-Rot Disease Using Zingiberaceae Essential Oils as Potential Weapons
Source: Front Plant Sci. 2018 Oct 4;9:1346. doi: 10.3389/fpls.2018.01346 (PMC6180181; doi:10.3389/fpls.2018.01346)
Supplement: Supplementary file 3 [file Image_1.pdf]

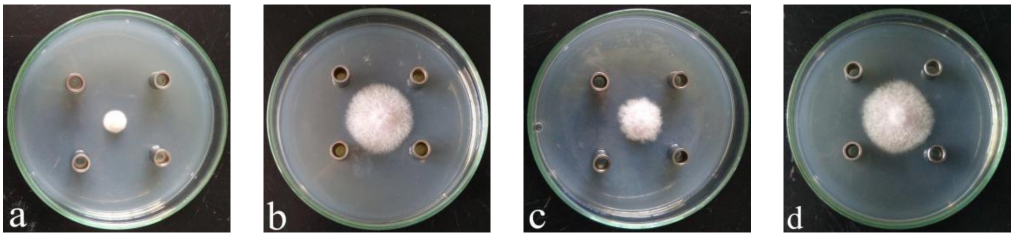

FIGURE S1 | Inhibitory effects of volatile or non-volatile portion of petroleum ether extract from *Z. officinale* on *F. oxysporum*. **(a)** Volatile portion, **(b)** Non-volatile portion, **(c)** Flutriafol as Positive Control, **(d)** 10/1000 DMSO and 1/1000 Tween 80 mixture were as Negative Control.
